# Sensitive detection of vortex-core resonance using amplitude-modulated magnetic field

**DOI:** 10.1038/srep17922

**Published:** 2015-12-09

**Authors:** Xiaomin Cui, Shaojie Hu, Makoto Hidegara, Satoshi Yakata, Takashi Kimura

**Affiliations:** 1 Graduate School of Information Science and Electrical Engineering, Kyushu University, 744 Motooka, Fukuoka, 819-0395, Japan; 2 Department of Physics, Kyushu University, 6-10-1 Hakozaki, Fukuoka 812-8581, Japan; 3 Department of Information Electronics, Fukuoka Institute of Technology, 3-30-1 Wajiro-higashi, Higashi-ku, Fukuoka, 811-0295 Japan; 4 Research Center for Quantum Nano-Spin Sciences, Kyushu University, 6-10-1 Hakozaki, Fukuoka 812-8581, Japan; 5 CREST, Japan Science and Technology Agency, Sanbancho, Tokyo 102-0075, Japan

## Abstract

Understanding and manipulating the dynamic properties of the magnetic vortices stabilized in patterned ferromagnetic structures are of great interest owing to the superior resonant features with the high thermal stability and their flexible tunability. So far, numerous methods for investigating the dynamic properties of the magnetic vortex have been proposed and demonstrated. However, those techniques have some regulations such as spatial resolution, experimental facility and sensitivity. Here, we develop a simple and sensitive method for investigating the vortex-core dynamics by using the electrically separated excitation and detection circuits. We demonstrate that the resonant oscillation of the magnetic vortex induced by the amplitude- modulated alternating-sign magnetic field is efficiently picked up by the lock-in detection with the modulated frequency. By extending this method, we also investigate the size dependence and the influence of the magneto-static interaction in the resonant property of the magnetic vortex.

Ferromagnetic materials with reduced dimensions show unique arrangements of magnetic spins by introducing the geometrically controlled magneto-static interactions. Such patterned domain structures are useful for application in future spintronic devices as well as for understanding of the fundamental spin-related physics^1^,^2^. Especially, the magnetic vortex structure, stabilized in a ferromagnetic disk with a diameter less than a micron, has a potential as a unit cell for high density magnetic storage because of negligible magnetostatic interaction^2^,^3^,^4^,^5^,^6^. In addition, the spin dynamics of magnetic vortices stabilized in micron or submicron scaled ferromagnetic disks are of particular interest because the wide range tuning of the eigenfrequency for the vortex can be realized by its sample dimension^7^,^8^,^9^,^10^. Moreover, its low frequency dispersion at the resonant oscillation with a high thermal stability is another great performance of the magnetic vortex^11^. Therefore, understanding the spin dynamics of the magnetic vortex is an important issue for further developments of the spin-related physics and its application.

So far, numerous methods for investigating the dynamic properties of the magnetic vortex have been proposed and demonstrated. Time resolved magneto-optical Kerr effect is a powerful technique to study ultrafast spin dynamics in ferromagnetic films and their patterned structures^12^,^13^. However, the spatial resolution is limited by the wave length of the laser beam, typically sub-micron range. Instead of the laser beam, soft X-ray microscopy enables us to image the detailed spin structures with sub-nanosecond time and nano-meter spatial resolutions^14^. However, this kind of experiment is only carried out under the limited conditions because of the large scale facility. Apart from the direct imaging technique, the detection of the magnetization dynamics using the electrical transport measurements are widely utilized. High frequency impedance measurements of a coplanar wave guide or microstrip line with nanomagnets using network analyzer enable to detect the translational resonant motions of the magnetic vortices^15^,^16^,^17^. However, because of the sensitivity of the inductive coupling, approximately 1000 nanomagnets are required for detecting the vortex dynamics. Homodyne detection technique using the magnetoresistance effects sensitively detects the dynamical vortex motion of a single ferromagnetic dot^18^,^19^,^20^. But its analysis is complicated because of the coexistence between the Oersted field and spin-transfer torque. In addition, the monolithic radio-frequency (RF) measurement schemes used in the homodyne electrical techniques regulate the situation of vortex excitation and prevent the detailed study of the dynamic characteristic. Sugimoto *et al*. developed another homodyne detection technique using two independent RF currents flowing in the excitation and detection circuits^21^. However, the RF current flowing in the detecting circuit may affect the vortex dynamics because the frequency of the detecting current is close to the resonant frequency. Here, by using an amplitude modulated RF magnetic field for exciting the vortex resonance, we develop a sensitive lock-in detection method for the vortex dynamics.

Figure 1 shows the schematic device structure for our measurement setup together with a scanning electron microscope image of part of the patterned circular disks. The sample consists of a chain of 40-nm-thick Permalloy (Py) dots connected by 200-nm-thick Cu pads and a periodical Cu electrode on the Py disks. Here, the number of the dot is 50, much smaller than that for network analyzer measurement^16^. The electrical connection between the Py dot and Cu electrode is insulated by a patterned SiO_2_ film whose thickness is 100 nm. The Py disks with diameters varying from 2 *μ*m to 4 *μ*m were fabricated by using a conventional lift-off method combined with the electron beam lithography. The Cu electrodes were deposited by a Joule-heat evaporator after the surface cleaning of the Py disk by a very low energy Ar ion milling. The electrical resistivity for the Py is 30 *μ*Ωcm, much larger than that for the Cu electrode (2.0 *μ*Ωcm)^22^. The anisotropic magnetoresitance (AMR) measurements under the magnetic field with the various direction were performed by two-terminal resistance measurement of 10 *μ*A. For the vortex dynamics measurement, the static magnetic field is applied along the horizontal (x) direction where the vortex core is shifted along y direction^23^. The resonant motions of the vortices are excited by injecting the RF signal into the Cu periodical electrode. The dynamic response of the vortices under the RF magnetic field with sweeping the frequency is monitored by measuring the voltage of the chain of the disk through the AMR change, as explained below.

Figure 2(a) shows the longitudinal and transverse AMR curves for the sample with the diameter of 2 *μ*m. In the measurements, the magnetic field does not exceed the annihilation field 20 mT, meaning that the vortex core is located in the disk during the measurement. As shown in Fig. 2(a), the field-dependence of the resistance is well fitted by the parabolic dependence. Here, the parabolic curve in the longitudinal AMR shows the horizontal shift. This is because the Cu electrode has a vertical offset from the center of the disk. By changing the direction of the magnetic field with assuming the linear field dependence of the core position under the low magnetic field^6^,^17^,^24^, we obtain the core-position dependence of the resistance as shown in Fig. 2(b).

To mathematically describe the position dependence of the resistance, we focus on the two-fold rotational symmetry originating from the anisotropic magnetoresistance. Besides, the resistance change due to the vortex core displacement along the *x* direction should be smaller than that along the *y* direction because the core displacement along the *x* axis is partially smeared out by the Cu pads. These features are surely confirmed in the experimental results. From these considerations with parabolic dependence of the resistance change on the core position shift, we found that the resistance change (from the origin (0, 0)) 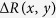 as a function of the two dimensional core position 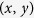 can be given by the following equation.


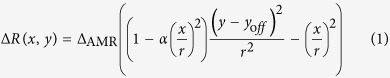


Here, 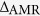 is the AMR change induced by the core displacement from the center to the position under magnetic field 10 mT along the *x* axis. *r* is the displacement of the vortex core due to the application of the magnetic field 10 mT along the *x* axis and *α* is the correction factor introduced by taking account of the influence of the Cu electrode for flowing dc current in the Py. We note that this description can be derived from the bivariate polynomial approximation. When the core is shifted to *x* direction from the center, the asymmetry with respect to the electrode increases. In such a situation, since the average y component of the magnetization becomes non zero, the resistance change due to the core displacement along *y* axis decreases compared to that at *x* = 0. Thus, the position dependence of the resistance is affected by the electrode. By fitting the experimental result shown in Fig. 2(b) to Eq. (1), the correction factor alpha for the present device is found to be 0.05. *y*_*off*_ is the offset due to the vertical shift of the Cu electrode. As seen in Fig. 2(c), the equation well reproduces the experimental results shown in Fig. 2(b).

We then consider the average resistance 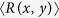 under the amplitude-modulated RF magnetic field with the static magnetic field along the *x* axis. The trajectory of the vortex core during resonant oscillation state induced by the RF magnetic field is known to be the circular shape not only in the absence of the magnetic field but also in the in-plane static magnetic field sufficiently smaller than the annihilation field^7^,^17^,^25^. Therefore, we adapt the following equations as the core trajectory under the amplitude-modulated RF magnetic field.


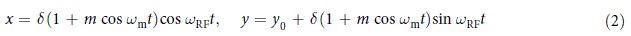


Here, 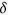 is the oscillation radius of the vortex core and its frequency dependence takes the Lorentzian resonant line shape. *m* is the modulation ratio and 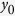 is the vertical shift of the core position due to the application of the horizontal static magnetic field. 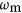 and 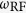 are the modulation and RF frequencies, respectively.

The induced voltage can be calculated by substituting Eq. (2) for Eq. (1) with multiplying 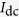. By using lock-in detection technique with 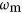, we can pick up only the coefficient for 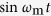 in the ac voltage. Since the experimentally detected voltage 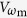 in lock-in amplifier is the time average, we obtain the following relationship for the effective resistance *R* between oscillation state and non-oscillation state (where *δ* is 0), which is defined by the average ac voltage 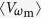 divided by 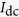.


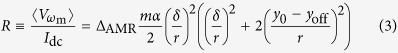


Here, The 1st term depends on the frequency but does not depend on the vertical shift 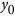. The 2nd term depends both on the frequency and the vertical shift. In the comparison between the 1st and 2nd terms, 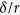, which is the relative oscillation amplitude, depends on the input RF power, but, in general, is smaller than 0.1 even at the resonant state^7^. However, 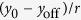 easily exceeds 0.1 by the application of the horizontal static magnetic field. Therefore, the 2nd term is dominant in Eq. (3). We emphasize that the detected voltage does not include any background signal, which is independent of the oscillation. This indicates that the present modulation method sensitively detects the voltage change due to the vortex oscillation.

To demonstrate the proposed technique for detecting the core resonance, we measure the effective resistance 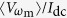 under the RF magnetic field. First, we measured the bias current dependence of the voltage spectra under the static magnetic field of 5 mT (Fig. 3(b)). Here, the dc current is varied from 1 mA to 20 mA. As can be seen in Fig. 3(b), we do not see any significant change of resonant frequency in this current range. The result indicates that the influence of the dc current on the vortex core resonance is negligibly small below 20 mA. This is consistent with the previous theoretical and experimental study^24^,^26^. From this consideration, we decided to use 6 mA, which is sufficiently small, in the series of the measurements presented below. Figure 3(c) shows the effective resistance as a function of the RF frequency at the fixed external fields of 0, 3.2 mT and 6.4 mT. Clear resistance dips 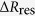 due to the vortex-core resonance are observed. Here, the magnitude of the dip 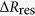 in the spectra is confirmed to increase with the static field. Moreover, as shown in Fig. 3(d), the field dependence of 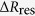 is well fitted by the parabolic equation. From the fitting, we find that 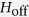 takes a positive value. This means that the number of the disks with CW chirality is larger than that with CCW chirality in this measurement. These experimental facts are highly consistent with the above expectation. We also point out that the resonant frequency is almost independent of the static magnetic field because the core potential is well expressed by the parabolic potential. This is clear evidence that a circular ferromagnetic disk creates a well-defined harmonic potential^17^,^27^. Moreover, we also plotted the resonant frequency as a function of the static magnetic field as indicated in Fig. 3(e). The resonant field shows the weak field dependence although the signature expected from the ideal parabolic potential should not depend on the magnetic field. The origin of this weak dependence may be related to nonlinear behavior of the resonant frequency and/or the formation of the anharmonic magnetostatic potential in the circular devices under a bias static field^28^.

We also perform the similar measurements for the chain of the disk with the different diameter. Figure 4(a) shows the spectra of the effective resistance 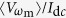 at *μ*_0_*H* = −4 mT for various disk diameters, 2 *μ*m, 3 *μ*m and 4 *μ*m. Here, the annihilation fields of the vortex for 2 *μ*m-, 3 *μ*m- and 4 *μ*m-diameter disks are about 20 mT, 18 mT and 16 mT, much larger than 4 mT. In each spectrum, a resistance dip due to the vortex resonance is clearly observed. The resonant frequencies for 2 *μ*m-, 3 *μ*m- and 4 *μ*m-diameter disks are 180 MHz, 135 MHz and 105 MHz, respectively, showing the monotonic increase with reducing the diameter of the disk. The observed size dependence of the resonant frequency is well reproduced by the numerical simulation using the object-oriented micromagnetic frame network (OOMMF). Numerically obtained resonant frequencies for 2 *μ*m, 3 *μ*m, and 4 *μ*m disks are, , respectively, 177 MHz, 132 MHz and 86 MHz, in consistent with the experimental results. Besides, we study the vortex dynamics for the magneto-statically coupled vortex system. As shown in the inset of Fig. 4(b), we have fabricated the chain of the magneto-statically coupled disks. Here, the disk diameter is 3 *μ*m and the edge-to-edge distance between the neighboring disk is 300 nm. Figure 4(b) shows the spectrum of the effective resistance 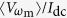 for the chain of the coupled vortices. The resistance dip due to the vortex resonance is clearly observed at 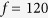 MHz, which is lower than that for the isolated disk with 3 *μ*m diameter shown in Fig. 3(c), suggesting an in-phase oscillation mode of the coupled vortices^29^,^30^,^31^. This excitation mode can be extended from the resonance in the pair of the coupled vortex dynamics with in-phase oscillation to a chain, which has been confirmed numerically and experimentally^8^,^29^,^32^.

We have developed a sensitive detection technique of the magnetic vortex resonance. Electrically separated excitation and detection circuits enable us to pick up the vortex oscillation without any background signal, resulting in the sensitive detection of the vortex resonance. The magnetic-field dependence of the resonant signal is well explained by the simple analytical model without considering spurious effects such as Oesrted field and spin-transfer torque. The obtained size and interval dependences of the resonant property show good agreement with the previously reported numerical and experimental results^7^,^18^. By extending the present technique to the application of the spatially modulated magnetic field, it is possible to excite the unique high-energy resonant mode^8^,^26^.

## Additional Information

**How to cite this article**: Cui, X. *et al.* Sensitive detection of vortex-core resonance using amplitude-modulated magnetic field. *Sci. Rep.* **5**, 17922; doi: 10.1038/srep17922 (2015).

## Figures and Tables

**Figure 1:**
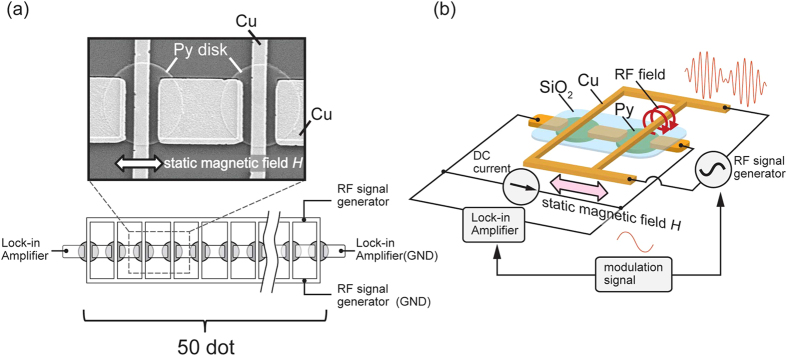
(**a**) Device configuration together with a SEM image of part of the device; (**b**) Schematic illustration of the proposed measurement setup for the vortex dynamics. During the measurement, an in-plane bias static magnetic field is applied parallel with the chain of Py disks. The amplitude modulated RF field is injected by flowing on the circuit of periodically patterned Cu electrode which locates on the top of the Py disk with an insulation layer of SiO_2_. The voltage change is monitored by another separated circuit combining the lock-in measurement system.

**Figure 2:**
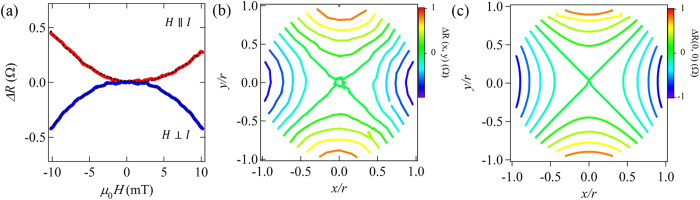
(**a**) Longitudinal and transverse AMR curves for the chain of the Py disks with 2 micron diameter. The AMR was measured using the traditional probe configuration for both 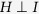 and 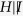, respectively. (**b**) Experimentally obtained resistance change (normalized) as a function of the core position. The AMR curve of the device was detected with changing magnetic field directions. Since the magnitude of the magnetic field is smaller than the annihilation field, we assume a linear field dependence of the core position. (**c**) Calculated position dependence of the resistance change from the origin (0, 0) based on Eq. (1) with *α* = 0.05.

**Figure 3:**
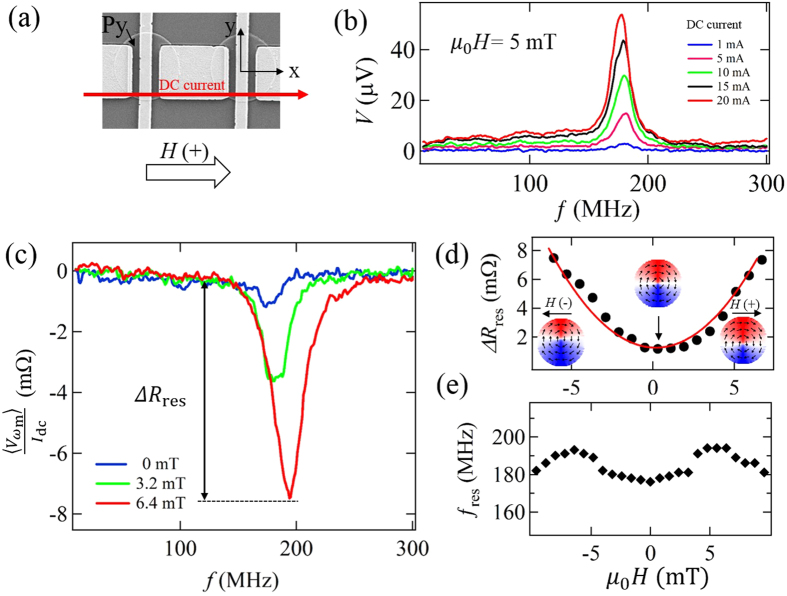
(**a**) Two experimental parameters; DC current 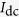 for generating the voltage including the resonant signature and static magnetic field *H* parallel with the dc current (*x* axis) for moving the core position along *y* axis. (**b**) Current dependence of the voltage spectra for the chained disk with the disk diameter of 2 *μ*m under the static magnetic field of 5 mT; (**c**) Frequency dependence of the average resistance change 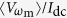 for the disk with 2 micron diameter for various static magnetic field. The voltage spectra were detected by flowing DC current of 6 mA with sweeping the RF frequency. (**d**) Field dependence of the resistance change 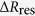 due to vortex core resonance. 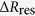 is defined as the resistance change between base line and resonance dip. The data is well fitted by a parabolic curve (solid line) based on Eq. (3). (e) The resonant frequency of the disk with 2 micron diameter as a function of bias static field. The frequency shows a weak dependence on the static magnetic field.

**Figure 4:**
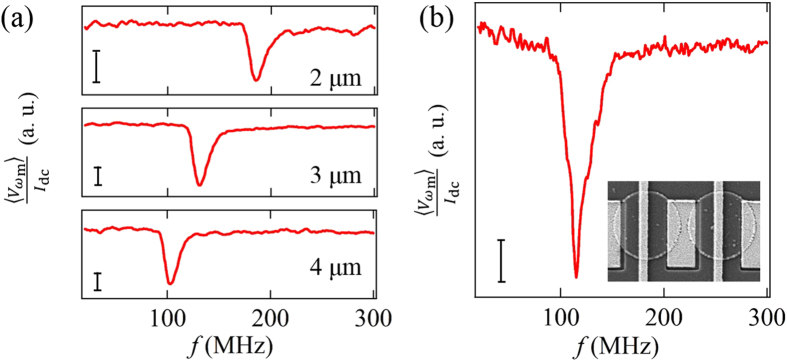
(**a**) 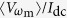 spectra at *H* = −4 mT for various disks with diameters of 2 *μ*m, 3 *μ*m and 4 *μ*m. The measurements have been carried out under the DC current of 6 mA and the RF amplitude of 5 dBm. (**b**) 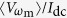 for the magneto-statically couple vortices with the diameter of 3 *μ*m. The inset shows a SEM image of the fabricated device where the edge-to-edge interval is 300 nm.
